# Belonging matters: The impact of social identification with classmates, friends, and family on interpersonal distance and bullying/cyberbullying in adolescence

**DOI:** 10.1371/journal.pone.0297370

**Published:** 2024-02-06

**Authors:** JuanJuan Wei, Michela Candini, Laura Menabò, Annalisa Guarini, Monica Rubini, Francesca Frassinetti

**Affiliations:** 1 Department of Psychology “Renzo Canestrari”, University of Bologna, Bologna, Italy; 2 Istituti Clinici Scientifici Maugeri IRCCS, Castel Goffredo, Italy; West University of Timisoara: Universitatea de Vest din Timisoara, ROMANIA

## Abstract

In adolescence individuals enlarge their social relationships and peer groups acquire a strong importance for their identity. Moreover, adolescents can experiment negative relationships with peers, i.e., bullying/cyberbullying. The present study aims to investigate the relationship between the feeling of belonging to a specific group, social identification, the distance that adolescents maintain interacting with others, interpersonal distance, and bullying/cyberbullying behaviors. Adolescents (age range 10–15 years) completed online measures of group identification (social identification with classmates, friends and family), interpersonal distance, and bullying and cyberbullying (perpetration and victimization). Results showed that adolescents with low social identification with classmates and friends chose larger interpersonal distance. Additionally, low scores in social identification with classmates were associated with higher victimization in cyberbullying. In contrast, adolescents with low scores in social identification with family were more involved as bullies in bullying and as victims in cyberbullying. Male adolescents were more likely to be victimized in bullying than females. This study underlines how social identification with peers and family works as a buffer in interfacing strangers, adjusting the distance maintained with them, and as a protective factor against aggressive relationships in adolescence. This study provides new opportunities for psychologists in understanding the psychological dynamics that shape social interactions among adolescents.

## 1 Introduction

Adolescence is a life period in which individuals enlarge their social world by becoming aware that they are members of multiple groups, such as their family, peer groups, and classmate groups [1]. The specific relationships that adolescents establish with these group members is likely to influence the social space they maintain between themselves and others during interactions, the so-called interpersonal distance [2–4]. Adolescence is also a life period in which individuals may experience negative and aggressive relationships with their peers, such as bullying and cyberbullying behaviors [5, 6]. Indeed, social identification plays a crucial role in shaping adolescents’ perceptions of their social environment and interpersonal behaviors [7]. It has been suggested that individuals who strongly identify with their group may exhibit a greater sense of connectedness and familiarity with group members [8]. Thus, social identification may impact how adolescents feel comfortable with others’ social proximity in everyday social interactions, leading to a reduction of interpersonal distance from them. However, given that social identification with one’s groups has many functions ranging from enhancement of self-esteem [9] to self-understanding [10], in this contribution we aim to tackle the impact of social identification with classmates, friends and family in influencing adolescents’ interpersonal distance with strangers, as well as how social identification can be related to perpetration and victimization in bullying and cyberbullying phenomena. By addressing the role of social identification with important groups of adolescents, we expect to offer guidance for future interventions targeting the ability to appropriately regulate interpersonal distance in different social environments, as well as practices aimed at contrasting bullying behavior consequences.

### 1.1 Social Identification

Social identification (SI) refers to the subjective aspects of group membership, which can lead to a sense of identity and self-definition together with feelings subjectively linked to a group [11, 12]. Moreover, as one of the critical social identity processes, social identification is conceptualized as determining the extent to which individuals behave in compliance with the behavioral norms of the groups to which they belong [13], and also a sense of emotional commitment to their groups [14]. Previous studies have highlighted that belonging to a social group has important implications for intergroup relationships and interpersonal behavior through social identification [15]. For instance, social identification is related to viewing the ingroup in a positive fashion compared to out-groups [9]. This has been confirmed by studies that consider SI in adolescence [16, 17]. Indeed, as adolescents explore their identity through social interactions, they realize to be members of various social groups ranging from their family [18, 19], to peer proximal groups (e.g. classmates and friends) and to more distal groups such as the human group [20]. Social identification, besides allowing to enhance group members’ self-esteem, allows them to experience other important functions such as intragroup comparison, self-understanding and leadership [10, 21].

As for the family group, high-quality family relationships can promote a healthy formation of the self and identity thus favoring adolescents’ harmonious development [18, 22]. Peer groups of classmates and friends are very important because adolescents spend most of their spare time outside the family [23–25]. Thus, they experience themselves in different roles by enacting possible selves and identities that they may then decide to choose. In this vein, interacting with peer group members not only prompts adolescents to experience reciprocity and intimacy, but also gives them an opportunity to perceive increased support and acceptance from their classmates and friends [26].

Considering the importance of classmates, friends and family in influencing adolescents’ values, attitudes and behaviors and in helping them to cope with adversities or set-backs, they might encounter [18, 20, 27], it is worth to investigate the impact of identification with these groups on actual social behavior. One of the core factors influencing social behavior is the distance that individuals maintain between themselves and others, the so-called interpersonal distance.

### 1.2 Interpersonal distance

Interpersonal distance (IPD) is the space around the body that individuals maintain between themselves and other people during social interactions [2–4], which implies that the regulation of IPD is grounded in actions that connect bodies in the environment. One of the main characteristics of IPD is its dynamicity: IPD is continuously regulated according to the variety of social environments and depends on one’s own feelings of comfort. The IPD is enlarged in hostile, threatening, and uncomfortable situations [28], whereas it is reduced in friendly, unthreatening, and comfortable situations [29]. This means that individuals can actively regulate their IPD by taking control of their environment.

Proxemics literature has demonstrated that IPD changes during the lifespan and can be modulated by individual differences of the interactants, such as age or gender [4, 30]. Indeed, IPD regulation is learned early in childhood and changes during adulthood [31]: as age increases, individuals take more distance from others [4, 32]. Thus, adolescents prefer a greater IPD than children and a shorter IPD than adults [31, 32]. As for the modulation of gender, adolescents usually maintain a smaller IPD from different-gender than same-gender individuals, especially males [33]. Moreover, considering same gender dyads, boys keep a larger distance from boys than girls from girls [34].

Interestingly, evidence on adult population showed a link between IPD and social identification [35, 36]: individuals preferred a larger distance from out-group compared to ingroup members [37]. For instance, in a competition, individuals preferred to seat close to a team-mate (ingroup member) rather than a competitor (outgroup member) [38]. In line, other studies put in evidence favoritism to in-group members and discrimination against outgroup members, especially when social identification is strong [39]. One of the possible forms of overt discrimination against out-group members among adolescents is bullying behavior [40].

### 1.3 Bullying and cyberbullying

Bullying is considered a specific type of aggressive behavior defined by three main components: repetition, imbalance of power and intention to harm [41]. In the last two decades, with the development of technology, a new form of bullying emerged, named cyberbullying, defined as an aggressive and deliberate behavior conducted by an individual or a group whose purpose is repeatedly and over time abusing a victim who cannot easily defend himself or herself, by using electronic devices [6, 42, 43]. Over the years, research has revealed that cyberbullying has unique features distinguishing it from traditional bullying, such as the absence of physical and temporal boundaries, enabling victims to be targeted at any time and place [44], and the ability to perpetrate aggression anonymously [45]. Additionally, cyberbullying allows for a potentially large audience, amplifying the impact and reach of aggression [46]. Nevertheless, bullying and cyberbullying are frequently seen as interconnected facets of the same phenomenon which has its roots in school and classroom dynamics [47–49]. Numerous studies have highlighted a significant overlap between these two forms of aggression [50, 51]. For instance, a study involving 2,028 Taiwanese students found that 48.7% of those engaged in cyberbullying were also involved in traditional bullying [52]. Similarly, in a comprehensive study by Cosma et al. (2020) that analyzed data from over 700,000 students across 37 countries in Europe and North America, an overlap was observed wherein 50% of the individuals who experienced cybervictimization also faced traditional bullying victimization [53].

Bullying and cyberbullying are both considered widespread problems that affect the well-being of adolescents on multiple levels, being associated with internalizing and externalizing symptoms, as shown by many empirical studies, meta-analyses and reviews [45, 54]. For example, Hawker and Boulton found in their meta-analysis that individuals who experienced bullying behaviors were more likely to have negative thoughts and depression symptoms [55]. Similar patterns were observed for cybervictimization and adolescents’ depression and life satisfaction [54, 56]. In addition, in the case of severe involvement, the risk of suicide is significantly higher [57].

Regarding gender, some differences in bullying and cyberbullying behaviors have been observed. Overall, a high rate of male perpetrators of bullying was revealed in many surveys [58, 59], whereas gender differences in victimization rates appeared to be less consistent [60–62]. Indeed, some studies found that males are more likely to be victimized than females [60, 62], while others reported that males experience less victimization than females [61]. Regarding cyberbullying, findings are mixed since research did not find a predominant gender involved either as a victim or as a bully [63, 64].

Some researchers have applied the social identification perspective to bullying phenomena, revealing that SI negatively correlates with bullying [40]. Individuals who strongly identify with their group are less likely to be targets of bullying, whereas individuals who are not perceived as part of one group are more likely to be bullied [65]. As for cyberbullying behavior, the stronger is the perceived peer-norm of behavior legitimacy within the ingroup, the higher the frequency of being involved in cyberbullying acts as perpetrators [66, 67]. This finding has the potential for developing preventive interventions targeting perpetration and victimization outcomes, by promoting individuals’ self-esteem and self-confidence as related outcomes of social identification and increasing the psychological resilience to bullying behavior [68, 69].

Since during adolescence there is an increasing social identification with peers rather than with family [70], and it is also the age when the risk of bullying and cyberbullying is highest [71], it is worthwhile to understand the role played by social identification with classmates, friends and family in preventing the frequency of bullying/cyberbullying behaviors during adolescence.

### 1.4 The present study

The first novelty of the present study is to investigate the influence of social identification on IPD regulation in adolescence, exploring whether IPD varies as a function of the level of identification with classmates, friends and family [12]. One could indeed argue that the more individuals are identified with significant groups, the less they need to keep distance from unknown others, since the psychological closeness to their groups may work as a protective factor in handling unknown people. Along this line, we tested whether social identification with classmates, friends and family, influences interpersonal distance. The second novelty is to examine whether bullying/cyberbullying behaviors vary depending on how adolescents identify with their classmates, friends and family groups.

Social identification was measured by the Scale of “Group Identification” (Identification with Classmates, Friends and Family) [12] and IPD was measured through an online modified version of the Interpersonal Visual Analogue Scale (IVAS) [30, 72] To assess bullying and cyberbullying phenomena, participants filled out two self-report questionnaires (European Bullying Intervention Project Questionnaire; European Cyberbullying Intervention Project Questionnaire) [73, 74].

We expect that adolescents with lower social identification should choose higher IPD and would be more involved in bullying and cyberbullying phenomena. Finally, accordingly with literature, we expect a significant effect of gender on IPD regulation and bullying and cyberbullying behaviors.

## 2 Methods

### 2.1 Participants

A priori power analysis was conducted on G*Power [75], revealing that 200 participants would yield 0.9 statistical power to detect a medium-size effect of 0.20 in a between-participants design. Considering possible drop-outs, a total of 242 students (111 males, age range = 10–15 years) attending secondary school and high school were recruited between March 2021 and May 2022 from the Emilia-Romagna region (Italy). Inclusion criteria were the following: i) age 10 to 15 years and ii) be free of any medical conditions that might interfere with the task. Therefore, since 38 participants did not complete the survey, the final sample consisted of a total of 204 participants (97 males, age range = 10–15 years; M±SD age = 12.36 ± 0.86 years, i.e., early adolescent and beyond).

The informed digital consent for participation in the study was provided by parents. In addition, as part of the survey, students were informed about the anonymous and voluntary nature of the survey, and the possibility of withdrawing from the study at any time. Recruitment and testing procedures were in line with the ethical standards of the Bioethics Committee of the Department of Psychology (Prot. n. 113714—University of Bologna) and the Declaration of Helsinki.

### 2.2 Materials and procedure

Qualtrics was used for the online survey (Qualtrics, Provo, USA). Participants sat alone in front of a computer screen and filled in an online questionnaire presenting consistently four sections in the same order: i) the European Bullying Intervention Project Questionnaire (EBIPQ) [74], ii) the European Cyberbullying Intervention Project Questionnaire (ECIPQ) [73], iii) the Interpersonal Visual Analogue Scale (IVAS) [30, 72], and iv) the Group Identification Scale [12]. The completion of the questionnaire took about 20–30 minutes.

#### 2.2.1 Social identification with classmates, friends, and family

Participants’ identification with classmates, friends, and family was measured by the Group Identification Scale [12]. Each subscale comprised 6 items with a response Likert-type ranging from 1 (strongly disagree) to 5 (strongly agree). These items capture cognitive, emotional and behavioral dimensions of participants’ SI: i.e., “Belonging to the group of my classmates/friends/family is very important for who I am”. High scores of social identification indicate that such groups are important to individuals’ self-definition [9]. For each context, Cronbach´s Alphas were also assessed (SI with classmates α = .87, SI with friends α = .83, SI with family α = .83).

#### 2.2.2 Interpersonal distance

**Fig 1:**
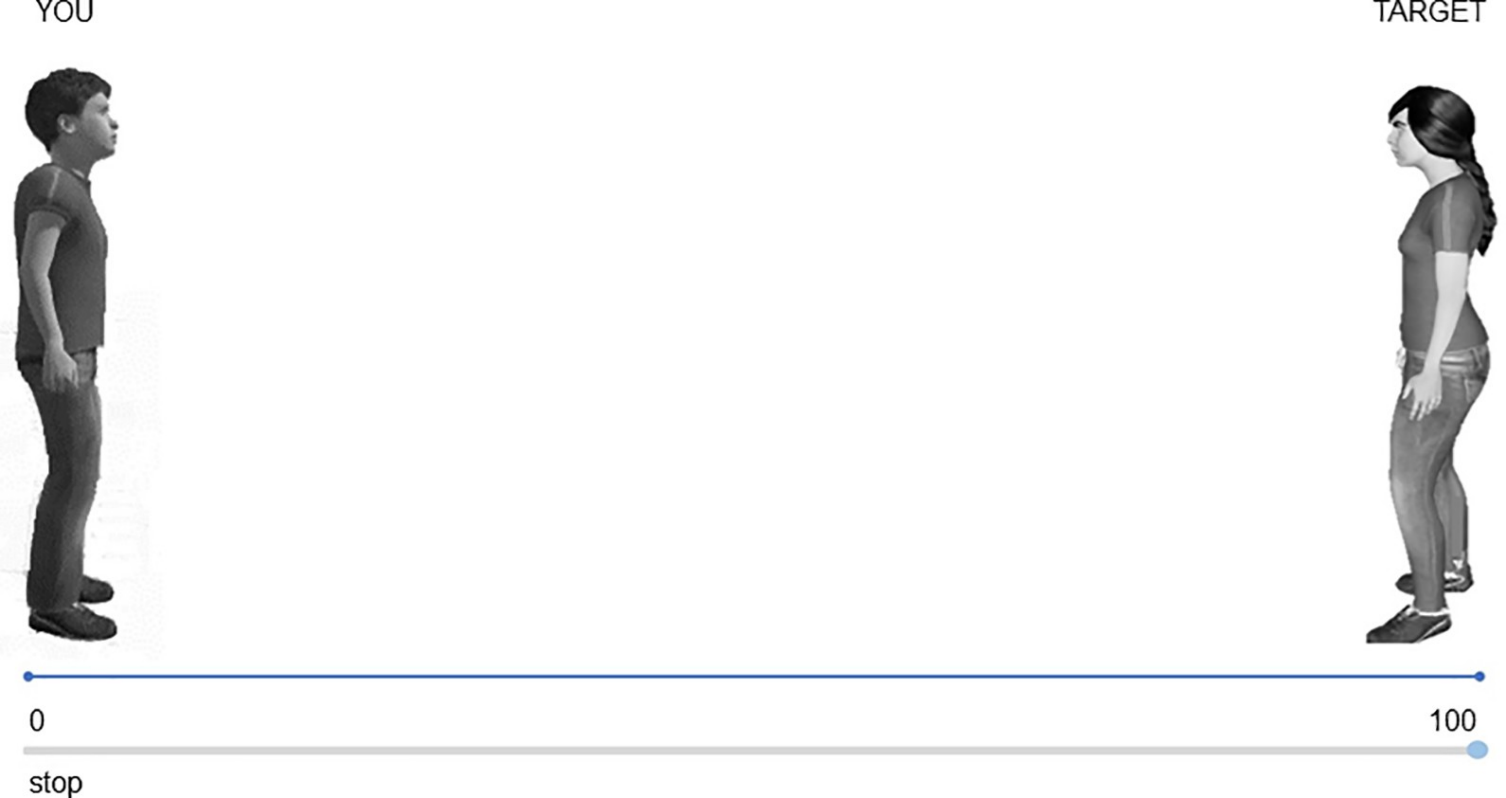
Example of IVAS trial. The participant is a male adolescent (YOU) and the actor on the opposite side of the line represents a female adolescent (TARGET). Participants chose their preferred IPD by moving the slider along the grey line (from 100 to 0): the further the slider was placed from the participant, the greater the distance from the target.

To measure preferred IPD we adopted the Interpersonal Visual Analogue Scale (IVAS) [30, 72], that was administered online. For each trial, a picture was displayed on a computer screen in which two different actors were depicted on the opposite side of a horizontal line: one actor represented the participant (labelled as “You”), and the other actor represented the target. The actor portraying the participant changed in accordance with his/her age (child or adolescent) and gender (male or female). Six different targets were presented: two children (male or female), two adolescents (male or female), or two adults (male or female). The actor’s starting position could be in front of or back to the participants. A total of sixteen trials were presented, and the order of the presentation was randomized across participants. The starting distance between the two actors was 100 mm. Participants were required to stand still and imagine the target walking toward them, and then they were asked to indicate, by moving a slider on the grey line, their preferred IPD from the target (from max 100 to min 0) (Fig 1). The greater the value chosen by the participant the larger the interpersonal distance maintained from the target.

#### 2.2.3 Bullying and cyberbullying

The European Bullying Intervention Project Questionnaire (EBIPQ) [74] consisted of 14 items, 7 for victimization and 7 for perpetration including physical bullying (i.e., “Someone has hit me”; “I hit someone”), social bullying (i.e., “Someone spread rumors about me”; “I spread rumors about someone else”), verbal bullying (i.e., “Someone insulted me”; “I insulted someone else”), and social exclusion (i.e., “I have been excluded or ignored by another person”; “I excluded or ignored another person”). Cronbach’s Alphas were calculated for each dependent variable (victimization α = .76; perpetration α = .83).

The European Cyberbullying Intervention Project Questionnaire (ECIPQ) [73] consisted of 22 items, along with two dimensions: cybervictimization (11 items) and cyberperpetration (11 items). The items describe different behaviors such as identity theft (i.e., “Someone has hacked into my account and pretended to be me”; “I hacked into someone else account and pretended to be it”), uploading or altering of awkward pictures or videos (i.e., “Someone has posted awkward images or videos of me on the Internet”; “I posted awkward images of videos of someone else on the internet”). Cronbach’s Alphas were assessed (cybervictimization α = .84; cyberperpetration α = .87).

In both questionnaires, participants had to indicate the frequency of each item using a 5-point Likert-type scale ranging from 0 to 4 (where 0 = never and 4 = more than once a week).

### 2.3 Data analysis

Data was analyzed by using SPSS Statistics 23 (IBM SPSS Statistics for Windows, Version 23.0. Armonk, NY: IBM Corp).

First, we conducted a Spearman’s correlation analysis, including all variables (e.g., social identification, IPD, bullying, and cyberbullying, see S1 Appendix). The relationships between social identification (identification with classmates, friends, and family) and IPD (expressed as the mean values obtained at the IVAS) are presented in the S1 Appendix.

To determine whether social identification influenced IPD, participants were categorized as low (n =  100; low-SI; *M* =  3.09 ± 0.46) or high in social identification (n =  104; high-SI; *M* =  4.05 ± 0.35) by using a median split (median value = 3.61). An independent sample t-test confirmed the significant difference between the two groups, *t*(202) = -16.49; *p* < .0001. The same median split procedure was adopted for each of the following social identification dimensions: SI with classmates (low-SI classmates: n =  87; *M* =  2.44 ± 0.54; high-SI classmates: n =  117; *M* =  3.74 ± 0.51; median value = 3.17; *t*(202) = -17.40; *p* < .0001), SI with friends (low-SI friends: n =  92; *M* =  2.99 ± 0.59; high-SI friends: n =  112; *M* =  4.13 ± 0.41; median value = 3.67; *t*(202) = -16.21; *p* < .0001) and SI with family (low-SI family: n =  88; *M* =  3.23 ± 0.49; high-SI family: n =  116; *M* =  4.46 ± 0.34; median value = 4.00; *t*(202) = -18.54; *p* < .0001). Then, we conducted three 2×2 Analysis of Variance (ANOVAs) on IPD mean scores with social identification (high and low SI) and gender of participant (male and female) as between-participants factors. Separate analyses were run for SI with classmates, SI with friends and SI with family.

Finally, the correlational relationship between SI, bullying (victimization and perpetration) and cyberbullying (cybervictimization and cyberperpetration) were depicted in the S1 Appendix. To assess whether social identification (SI) impacts on the experience of bullying and cyberbullying among adolescents, separate ANOVAs were conducted on mean scores of victimization, perpetration, cybervictimization and cyberperpetration, with SI (high and low SI) and gender of participant (male and female) as between-participants factors. Separate analyses were run for SI with classmates, SI with friends and SI with family.

Equal variances across samples have been assessed by using Levene’s Test (all variables conform to homogeneity of variance, except for the victimization variable: *p* = .010). Bonferroni’s correction was adopted and the partial eta-squared (η^2^_p_) indicated the effect size.

## 3 Results

### 3.1 Social identification and gender of participant on IPD

**Fig 2:**
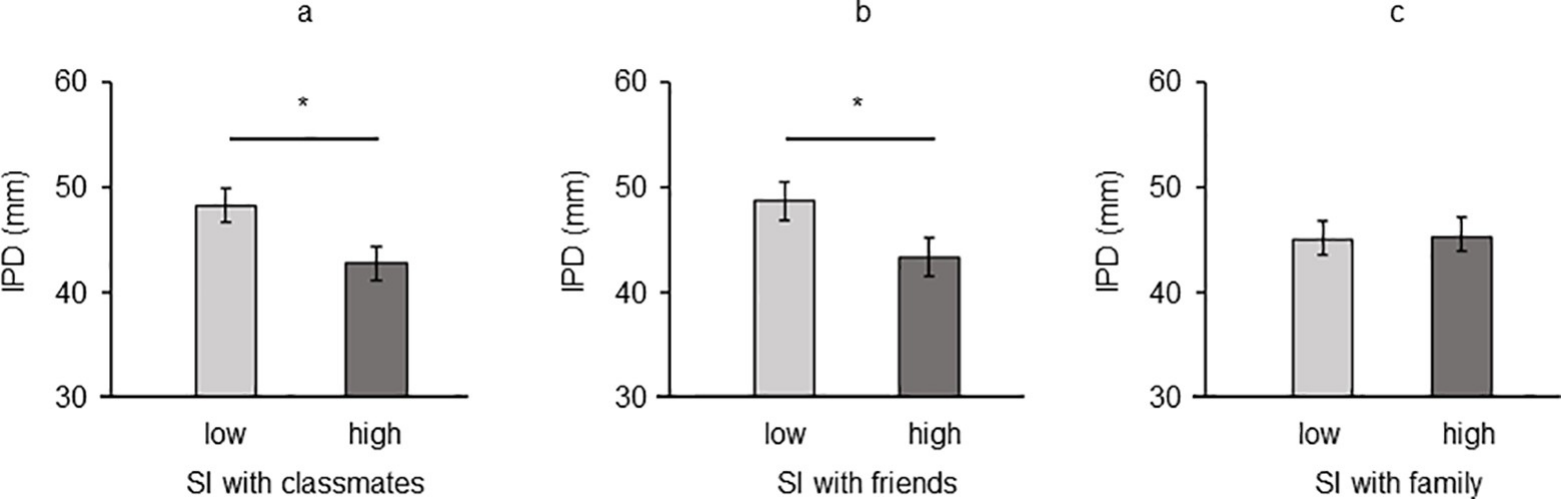
IPD as a function of (a) Social Identification with Classmates, (b) Social Identification with Friends and (c) Social Identification with Family. Error bars indicate standard deviation (SD). Asterisks reveal significant differences (*p* < .05).

The ANOVA showed a significant effect of SI with classmates on IPD [*F*(1, 200) = 6.049, *p* = .015, η^2^_p_ = .029]: a larger IPD was found among those adolescents reporting low SI with classmates (*M* = 48.21, *SEM* = 1.70) compared to those with high SI with classmates (*M* = 42.69, *SEM* = 1.46, Fig 2A). A significant main effect of SI with friends on IPD was also obtained [*F*(1, 200) = 5.066, *p* = .025, η^2^_p_ = .025]: a larger IPD was found among those with low SI with their group of friends (*M* = 47.76, *SEM* = 1.65) compared to those with high SI with the group of friends (*M* = 42.72, *SEM* = 1.51, Fig 2B). No significant effect of SI with family on IPD was found (*p* = .17; Fig 2C). Gender of participant (all *ps* > .531) and its interaction with SI variables were not significant in all the analysis conducted (all *ps* > .588).

### 3.2 Social identification and gender of participant on bullying

A main effect of Gender of participant was revealed on victimization, as male participants were more victimized than female participants. This result was consistent in all analyses involving SI with classmates [*F*(1, 200) = 5.105, *p* = .025, η^2^_p_ = .025], SI with the family [*F*(1, 200) = 4.684, *p* = .032, η^2^_p_ = .023], and SI with friends [*F*(1, 200) = 3.671, *p* = .057; η^2^_p_ = .018, tendency]. SI with classmates, friends, and family (all *ps* > .116) and its interaction with gender (all *ps* > .235) were not significant on victimization.

**Fig 3:**
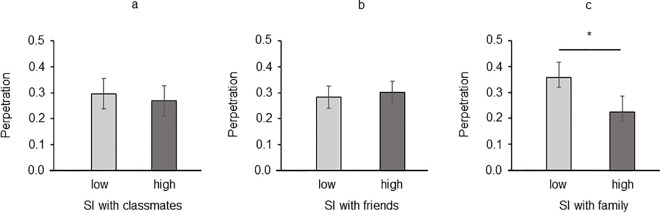
Perpetration mean scores as a function of (a) Social Identification with Classmates, (b) Social Identification with Friends, and (c) Social Identification with Family. Error bars indicate standard deviation (SD). Asterisks reveal significant differences (*p* < .05).

Concerning perpetration, the variable SI with the family revealed a significant main effect [*F*(1, 200) = 4.270, *p* = .040, η^2^_p_ = .021]: adolescents with low SI with their family (*M* = 0.363, *SEM* = 0.051, Fig 3C) were more involved in perpetration compared to adolescents with high levels of SI (*M* = 0.224, *SEM* = 0.044). By contrast, SI with classmates [*F*(1, 200) = 0.205, *p* = .651, η^2^_p_ = .001; Fig 3A] and SI with friends [*F*(1, 200) = 0.158, *p* = .692, η^2^_p_ = .001] were not significant (Fig 3B). Gender of participant (all *ps* > .081) and its interaction with SI variables were not significant (all *ps* > .669).

### 3.3 Social identification and gender of participant on cyberbullying

**Fig 4:**
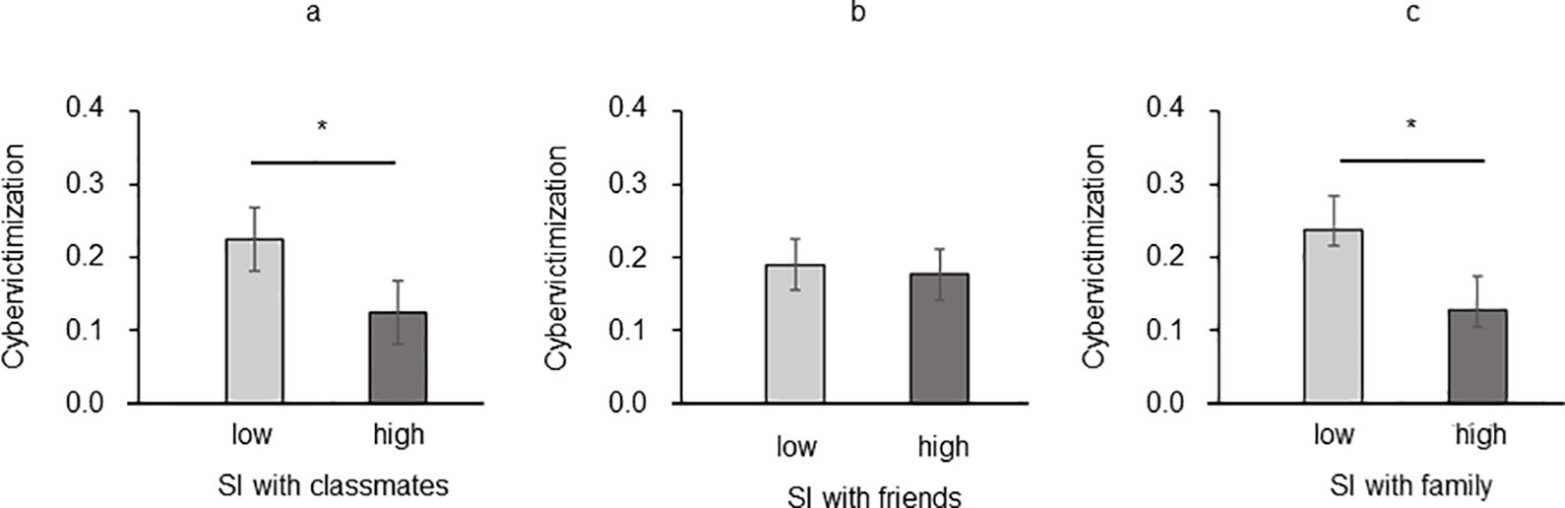
Cybervictimization mean score as a function of (a) Social Identification with Classmate, (b) Social Identification with Friends, and (c) Social Identification with Family. Error bars indicate standard deviation (SD). Asterisks reveal significant differences (*p* < .05).

SI with classmates showed a significant main effect on cybervictimization [*F*(1, 200) = 4.521, *p* = .035, η^2^_p_ = .022] due to lower cybervictimization among adolescents with high SI with classmates (*M* = 0.124, *SEM* = 0.03) than in adolescents with low SI (*M* = 0.224, *SEM* = 0.036, Fig 4A). SI with friends was not significant [*F*(1, 200) = 0.059, *p* = .808, η^2^_p_ = .001] (Fig 4B), while SI with family revealed a significant main effect [*F*(1, 200) = 5.120, *p* = .025, η^2^_p_ = .025] due to lower cybervictimization among adolescents with high SI with their family group (*M* = 0.135, *SEM* = 0.028) than in adolescents with low family SI (*M* = 0.227, *SEM* = 0.032, Fig 4C). Gender of participant (all *ps* > .675) and its interaction with SI variables were not significant (all *ps* > .554).

As for cyberperpetration, social identification (all *ps* > .069), gender of participant (all *ps* > .315) and their interactions were not significant (all *ps* > .731).

## 4 Discussion

The present study aimed at exploring how social identification (SI) with meaningful social groups (i.e., classmates, friends and family) to which early adolescents and beyond belong, influences the choice of interpersonal distance (IPD) measured in a computerized task (i.e., IVAS) [30, 72]. Moreover, considering the relationship between peer group membership and aggressive behaviors among peers [67, 76, 77], we explored the influence of social identification on perpetration and victimization in bullying and cyberbullying phenomena.

Findings showed that SI with classmates and friends influences IPD chosen by participants: adolescents with higher social identification chose shorter interpersonal distance, and this is consistent with our hypothesis. This finding may imply that social identification, especially with peers, provides adolescents with a certain degree of confidence, trust and control over their social world, leading them to choose a short interpersonal distance, even with strangers. Even if it is known from previous research that IPD changes in accordance with the social context in which individuals interact [78], the novelty of the present study relies on unfolding the role of social identification in regulating IPD, behaviorally measured in a computerized task. Indeed, IPD is enlarged in threatening and uncomfortable situations, whereas it is reduced in unthreatening and comfortable situations [28, 29, 79, 80]. One comfortable and friendly situation experienced by adolescents is when they share a sense of identification and goals with significant peers. Since they spend most of their time at school, they can experience a sense of inclusivity and reciprocal trust with classmates and friends, very likely leading to develop a high social identification with them. In this vein, adolescents who strongly identify with their peer groups may choose close proximity with others, even if those are unfamiliar, likely because they perceive a high sense of security. In this respect, our results not only confirm the pivotal role that social identification plays in regulating social behavior [81], but also substantially extend the functions of social identification, which mainly emphasize individual needs (i.e., self-insight and understanding, leadership role, romantic relations) and as well as group-relevant motivations (i.e., ingroup cohesion, inter-group comparison and competition) [10].

We found interesting results concerning the influence of specific dimensions of social identification on the perpetration and victimization of bullying and cyberbullying. Concerning traditional bullying, adolescents with lower social identification with family enacted more aggressive behaviors. This evidence indicates the crucial role of the parent-child relationships in influencing the chances of being engaged in bullying episodes [82, 83]. Over the years, bullying perpetration was related to low parent-child involvement [84], and to the presence of negative parents’ emotions [85, 86]. For example, Bibou-Nakou and colleagues (2013) found that bullying behavior was influenced by poor relationships with parents, including a lack of warmth and empathy from them [87]. Additionally, previous research showed that bullies and victims adopt less open and more offensive communication with parents than adolescents who are not involved [88]. Our study adds to the previous literature the specific role of social identification with one’s family in association with bullying perpetration, suggesting that family constitutes the primary social context where adolescents learn how to manage interpersonal aggressiveness and conflicts [89].

Concerning cyberbullying, we found an important role of social identification with family and classmates in cybervictimization. In other words, adolescents with a low social identification with their family, but also with peers in their class, reported more experiences of cybervictimization. As for social identification with the family, our results resonate with previous findings that identified issues with parents as a major risk for being victimized/cybervictimized [90]. For example, children with divorced/widowed parents were more likely to become cybervictims [91]. Moreover, Larrañaga et al. (2016) found that cybervictims avoid communication with parents, which contributed to the lengthening of the cybervictimization’s duration [92].

Concerning social identification with classmates, our research underscores the pivotal role that this social group plays also in online aggressive dynamics, aligning with prior studies in this domain. For instance, Pyżalski and colleagues’ qualitative analysis (2022) highlighted that, although bullying often migrates to the digital realm, it predominantly occurs within circles of classmates [49]. Similarly, a recent study by Menabò et al. (2023) revealed the critical importance of peer networks, not only in cases of victimization but also in cybervictimization [48].

Indeed, the association between victimization and low social identification with classmates revealed that if a group member establishes a low identification with one’s own group, he or she may be excluded, especially in friendship groups where the other members may be strongly bounded to each other through social identification both in an online and offline context. Notably, being highly identified with classmates works as a buffer against cybervictimization [47]. Thus, accordingly with the protective function of high social identification, adolescents may be more committed to their class and prevent each other from being victimized [93].

Regarding the role of gender, we did not find a significant effect on perpetration of bullying and cyberbullying. However, we found that males were more frequently victimized than females in traditional bullying. This is consistent with those previous studies showing that males are more likely to be exposed to the experience of victimization [94, 95]. However, as already mentioned in the introduction there are some mixed results in the literature regarding the role of gender [60–62, 93], which requires further examination. Concerning cyberbullying, the lack of gender differences is in line with previous findings, which have shown that cyberbullying is not a gender-specific behavior [96].

Overall, the results of the study underline how social identification with peers and family is a buffer for interfacing with others, through adjusting the distance we maintain with them, and as a protective factor against transgressive behaviors in adolescence.

### 4.1 Limitations and future directions

The current study has some limitations that should be considered when interpreting the results. First, our findings are limited to a specific age range (i.e., 10–15 years). How the impact of social identification on interpersonal distance regulation, and bullying/cyberbullying behaviors change over time should be further investigated by longitudinal studies. Secondly, the sample size in our study should be taken into account considering the generalizability of our findings to other populations. Therefore, future research with larger and cross-cultural samples is needed to increase generalizability and external validity of the results.

In addition, although the present research marks an initial step towards understanding the relationship between social identification processes, interpersonal distance, bullying and cyberbullying, the cross-sectional design of the study provides a static representation of relationships. Future research could provide deeper insights into these relationships, explaining the processes over time. Indeed, different processes could come into play. For example, the chosen IPD may serve as a non-verbal cue and may be a critical indicator of an individual’s sense of connectedness/disconnectedness from others. Previous studies [97] indicate that individuals who experience bullying tend to display more withdrawn behaviors compared to their non-bullied peers. This tendency towards withdrawal might be reflected in a preference for maintaining a greater interpersonal distance. At the same time, however, the choice of a larger interpersonal distance may imply a lack of interest or engagement with peers, potentially leading to or exacerbating social exclusion. To fully unravel these complex interactions, future studies should aim to track these dynamics over time, potentially revealing critical patterns and causal links between the choice of interpersonal distance, social identification processes, and the cycle of bullying and cyberbullying.

## 5 Conclusions and implications

The current findings show the importance of social identification with peers and family in regulating interpersonal distance and preventing bullying and cyberbullying behaviors in early adolescence and beyond. This evidence sheds new light on the understanding of these phenomena that can also inform psychologists and educators in providing guidance to adolescents in their adaptation to school and life challenges. For example, educators can implement class-building activities or programs that promote class cohesion and foster a sense of connectedness and solidarity with students in a welcoming and inclusive environment. Indeed, schools are a privileged setting for interventions since they provide the opportunity for building a sense of shared identity and group common goals. Therefore, students are more likely to feel comfortable interacting with strangers and choose optimal interpersonal distances [8, 98].

Furthermore, our results demonstrate the central role that the family plays in traditional perpetration dynamics. In this regard, parents/caregivers can benefit from interventions designed to improve the relationship with their offspring, including improving communication with their children and taking an empathic perspective [99, 100]. Moreover, low social identification with parents and classmates can increase the risk of becoming a cybervictim. Therefore, parents and teachers may benefit from being trained about online mediation strategies to protect adolescents and teach them how to safely navigate online [101, 102].

In conclusion, the gathered evidence shows that a fundamental social psychological factor such as social identification with peers and family by influencing interpersonal distance with strangers and avoidance of bully behaviors very likely helps to pave the way for a robust adaptation and mental health of individuals [11, 103]. Future research is needed to provide evidence on this consequence.

## Supporting information

S1 Appendix (DOCX)
