# Supplementary material for: Belonging matters: The impact of social identification with classmates, friends, and family on interpersonal distance and bullying/cyberbullying in adolescence
Source: PLoS One. 2024 Feb 6;19(2):e0297370. doi: 10.1371/journal.pone.0297370 (PMC10846719; doi:10.1371/journal.pone.0297370)
Supplement: S1 Appendix — (DOCX) [file pone.0297370.s001.docx]

**Supporting Information**

**Appendix**

**Correlations between Social Identification and IPD**

Social identification (SI) with classmates (*r* = -.254, *p* < .01) and SI with friends (*r* = - .159, *p* = .04) negatively correlated with preferred interpersonal distance (IPD): the less adolescents think of themselves as a member of their friends or classmates’ group, the larger IPD they choose. No significant correlation was found between SI with family (*r* = -.010, *p* = .83) (Table 1).

**Correlations between Social Identification and Bullying/Cyberbullying**

Considering traditional bullying, significant negative correlations emerged between victimization and SI with classmates (*r* = -.176, *p* = .012): adolescents with high scores in social identification with their classmates showed a low victimization rate. Moreover, a significant negative correlation between perpetration and SI with family was revealed (*r* = -.166, *p* = .018): the more adolescents perceived themselves as members of their family, the less they perpetrated aggressive behaviors. Correlations between SI with friends and victimization and perpetration were not significant (*p* > .140).

In a similar vein, looking at the cyberbullying phenomenon, significant negative correlations were found between SI with classmates and cybervictimization (*r* = -.186, *p* = .008): the high the SI with the classmates, the low the cybervictimization. No significant correlations between SI with friends and SI with family and cyberbullying were found (all *ps* > .069).

**Table 1. Spearman’ Correlations among Interpersonal Distance (IPD), Social Identification (SI), and Bullying/cyberbullying**

| Variable | IPD | SI with classmates | SI with friends | SI with family | | Victimization | Perpetration | Cybervictimization | Cyberperpetration |
| --- | --- | --- | --- | --- | --- | --- | --- | --- | --- |
| IPD | 1.000 |  |  |  |  | |  |  |  |
| SI with classmates | -.254^**^ | 1.000 |  |  |  | |  |  |  |
| SI with friends | -.159^*^ | .547^**^ | 1.000 |  |  | |  |  |  |
| SI with family | -0.010 | .328^**^ | .393^**^ | 1.000 |  | |  |  |  |
| Victimization | -0.093 | -.176^*^ | -0.104 | -0.104 | 1.000 | |  |  |  |
| Perpetration | 0.009 | -0.049 | -0.039 | -.166^*^ | .649^**^ | | 1.000 |  |  |
| Cybervictimization | 0.031 | -.186^**^ | -0.052 | -0.120 | .616^**^ | | .490^**^ | 1.000 |  |
| Cyberperpetration | 0.056 | -0.052 | -0.006 | -0.127 | .471^**^ | | .619^**^ | .625^**^ | 1.000 |

**p* < .05; ***p* < .01; ****p* < .001
